# Computational refinement of spectroscopic FRET measurements

**DOI:** 10.1016/j.dib.2017.03.041

**Published:** 2017-04-02

**Authors:** Alexander Kyrychenko, Mykola V. Rodnin, Chiranjib Ghatak, Alexey S. Ladokhin

**Affiliations:** a Institute of Chemistry and School of Chemistry, V. N. Karazin Kharkiv National University, 4 Svobody Square, Kharkiv 61022, Ukraine; b Department of Biochemistry and Molecular Biology, The University of Kansas Medical Center Kansas City, KS66160-7421, USA

**Keywords:** FRET, Fluorescent protein, ECFP, EYFP, Single molecule, Molecular dynamics simulations

## Abstract

This article supplies raw data related to a research article entitled “Joint refinement of FRET measurements using spectroscopic and computational tools” (Kyrychenko et al., 2017) [1], in which we demonstrate the use of molecular dynamics simulations to estimate FRET orientational factors in a benchmark donor-linker-acceptor system of enhanced cyan (ECFP) and enhanced yellow (EYFP) fluorescent proteins. This can improve the recalculation of donor-acceptor distance information from single-molecule FRET measurements.

**Specifications Table**

**Table:** 

Subject area	*Chemistry, Biophysics*
More specific subject area	*Computational refinement of Förster resonant energy transfer*
Type of data	*Figure, text*
How data was acquired	*Computer simulations using molecular dynamics method*
Data format	*Raw, analyzed, processed*
Experimental factors	*Fluorescence measurement procedure, molecular dynamics simulation details*
Experimental features	*MD simulations details*
Data source location	*Kansas City, USA; Kharkiv, Ukraine*
Data accessibility	*Data given as Supplemental files in this article*

**Value of the data**

• Details and flowchart of MD simulations of conformational sampling of ECFP and EYFP proteins linked with a flexible unstructured Gly/Ser peptide are provided.
• MD simulation setup and force field parameterization of ECFP and EYFP are given.
• MD sampling performed in the absence and in the presence of 0.1 M buffering ions of Na^+^ and Cl^-^ revealed that the increased ionic strength had no effect on the equilibrium distance distributions between EFCP and EYFP moieties.
• The distribution of the FRET orientational factor, *κ*^2^, estimated from MD simulations, is found to correspond well to the theoretical curve for an ideal, unrestricted, isotropic re-orientation of dipoles.
• The comparison of the effect of the standard assumption about the unique value of the Förster radius R_0_ on the accuracy of determination of the Donor-to-Acceptor distance distributions is presented, revealing that both *apparent* distributions calculated from either MD-generated or experimentally measured FRET efficiencies systematically overestimate the width of the *true* distance distribution *R*_DA_.

## 1 Data

**Fig. 1:**
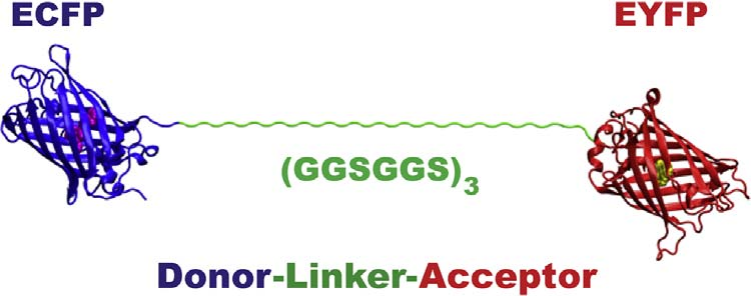
Scheme of an ECFP-donor and EYFP-acceptor FRET pair linked by a flexible peptide bridge (GGSGGS)_3_ referred to as ECFP-l_3_*-*EYFP.

MD simulations for a benchmark donor-linker-acceptor system composed of enhanced cyan (ECFP) and enhanced yellow (EYFP) fluorescent proteins were used to estimate FRET orientational factors, which can improve the recalculation of donor–acceptor distance information from sm-FRET measurements. ECFP and EYFP proteins were linked with a flexible unstructured Gly/Ser peptide linker composed of 3 repeating units of GlyGlySerGlyGlySer (GGSGGS) as shown in Fig. 1.

### 1.1 MD sampling of ECFP-l_3_-EYFP by multiple discrete MD simulations

Proper conformation sampling of l_3_ linker is essential for the accurate analysis of MD simulations in ECFP-l_3_-EYFP. In general, the convergence of the MD sampling of long-chain polypeptides is challenging, because they have multidimensional energy surfaces characterized by many local minima separated by potentially high free-energy barriers, which can lead to kinetic trapping. In our system, however, we did not expect MD sampling to become restricted to a series of localized metastable conformations, because the flexible linker connecting two rigid FPs is not expected to create high-energy barriers. Nevertheless, in order to ensure sufficient conformational MD sampling, we carried out a series of multiple discrete MD runs, each initiated with random initial structures. Such an approach has been shown to enhance conformational sampling [2], [3], [4], [5]. To investigate the role of starting configurations in the equilibrium structure of EFCP-l_3_-EYFP, we used a series of four independent parallel MD simulations, each of which was carried out with a different initial conformation of l_3_ linker.

**Fig. 2:**
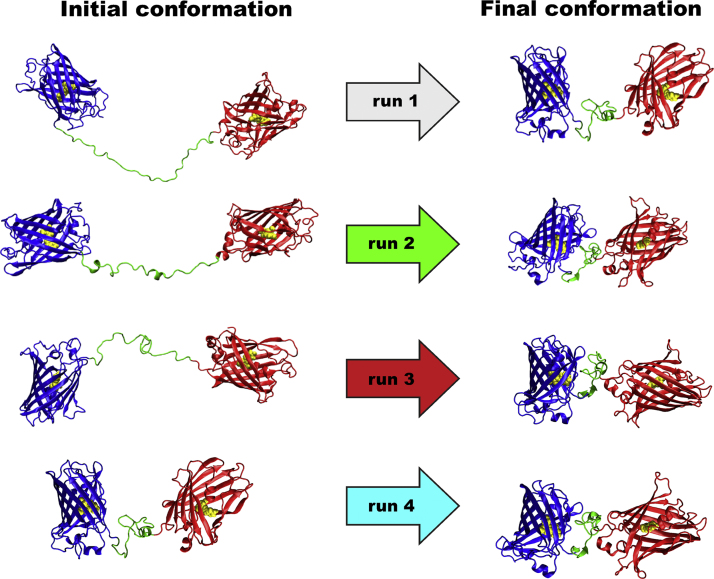
(**A**) The series of the four different starting configurations of ECFP-l_3_-EYFP (runs 1–4) were run in parallel to achieve adequate convergence statistics. (*Left*) MD snapshots of the initial conformations of ECFP-l_3_-EYFP. (*Right*) MD snapshots of final equilibrium conformation of ECFP-l_3_-EYFP, taken at the end of the MD sampling.

**Fig. 3:**
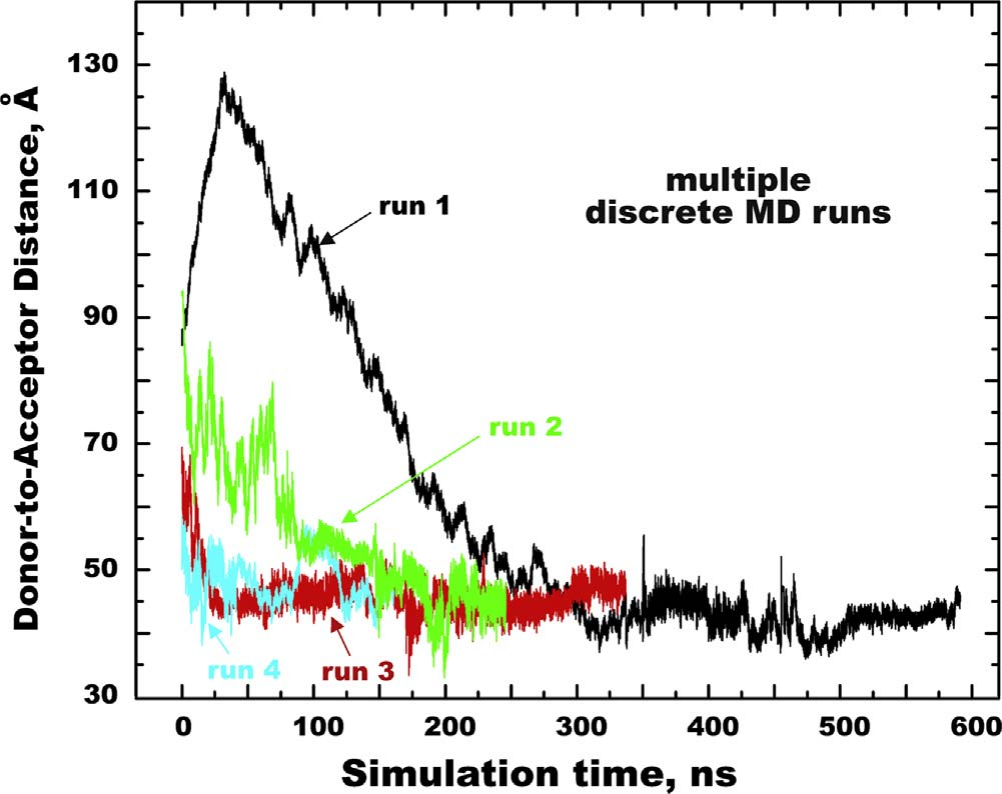
Parallel MD sampling of ECFP-l_3_-EYFP. The series of the four different starting configurations of ECFP-l_3_-EYFP (run 1–4) were run in parallel to achieve adequate convergence statistics.

Fig. 2 show MD snapshots of the initial and final conformations of EFCP-l_3_-EYFP for the four different MD runs referred to as runs 1–4. In runs 1–2, EFCP-l_3_-EYFP was constructed as having the extended conformation of l_3_, so that the initial value of the donor-to-acceptor distance *R*_DA_ was about 90 Å. In runs 3–4, the EFCP and EYFP moieties were set in different orientations to avoid local conformation trapping during MD sampling. The large-scale MD parallel simulations of runs 1–4 revealed that in all studied systems *R*_DA_ converged to some plateau at ~42–46 Å (Fig. 3). From these MD runs the last 100–200 ns were used for the analysis, during which the average orientation factor approached the theoretical value for unrestricted diffusion, confirming that the time range of our simulations is sufficient for the proper conformational sampling of the studied system.

### 1.2 Effect of ionic strength

**Fig. 4:**
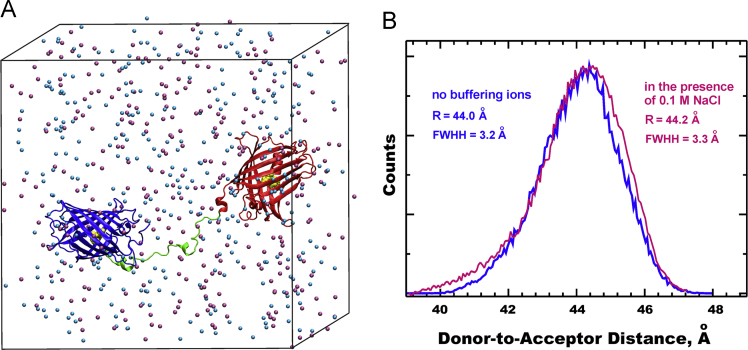
MD simulations of ECFP-to-EYFP distance. (**A**) Snapshot of ECFP-l_3_-EYFP in a MD box in the presence of 0.1 M buffering ions Na^+^ (*cyan balls*) and Cl^−^ (*mauve balls*). (**B**) Comparison of ECFP-to-EYFP distance distributions calculated in the absence (*blue*) and in the presence of 0.1 M NaCl (*magenta*).

Most MD samplings were carried out in water with sodium ions added to counterbalance the protein charge. To estimate the role of ionic strength in conformation equilibrium of EFCP-l_3_-EYFP, the control MD simulation (100 ns) was also performed in the presence of 0.1 M buffering ions of Na^+^ and Cl^-^ (Fig. 4A), which revealed only a small effect of ionic strength on equilibrium distances between bulk EFCP and EYFP moieties (Fig. 4B).

### 1.3 Orientation factor *κ*^*2*^

**Fig. 5:**
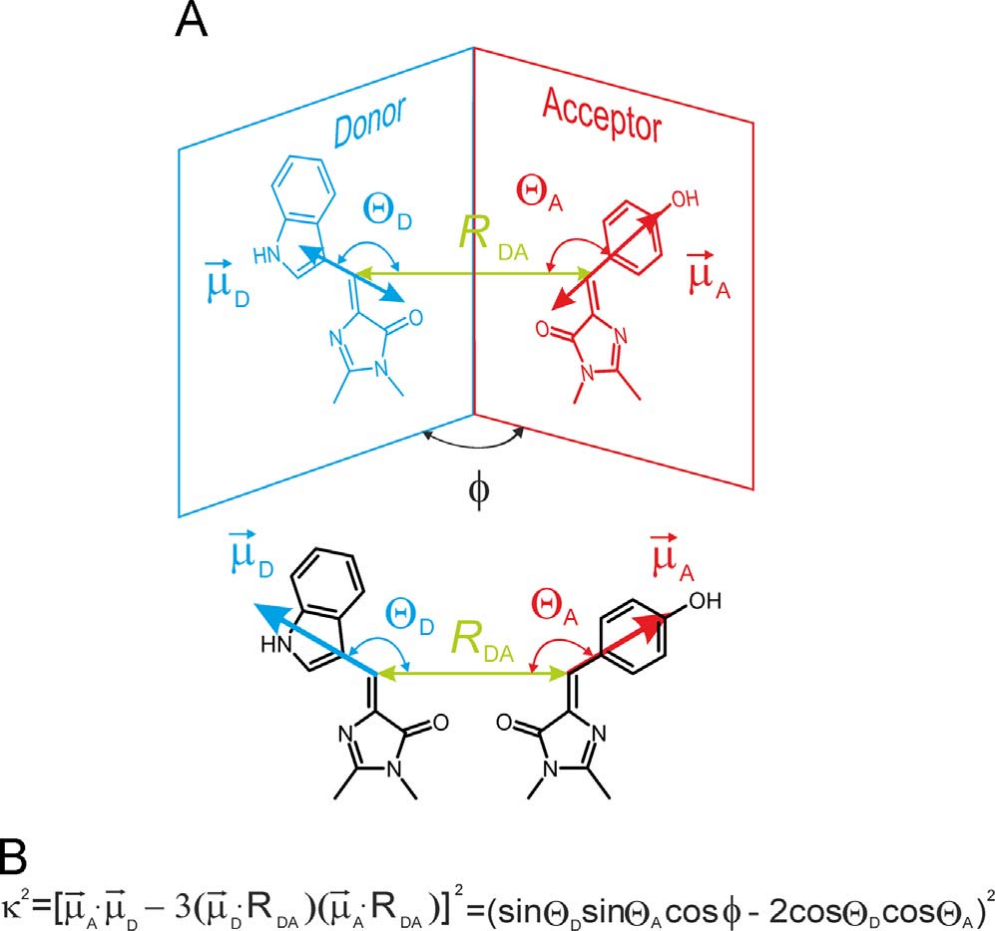
(**A**) The transition dipole moments of the ECFP donor and EYFP acceptor chromophores are shown as vectors µ_D_ and µ_A_, respectively. The angles Θ_D_ and Θ_A_ define the dipole orientations with respect to the connecting unit distance vector *R*_DA_, whereas *φ* is the angle between the two planes. (**B**) The definition of the orientation factor *κ*^2^.

The orientation factor *κ*^2^ that provides the dependence of the interaction between two electric dipoles on their orientations as shown in Fig. 5. *κ*^2^ can be defined as shown in Fig. 5B. To calculate instantaneous *κ*^2^(*t*) from MD trajectories, the angles *θ*_D_, *θ*_A_ and distance *R*_DA_ were calculated by using GROMACS analysis utilities *g_angle* and *g_sgangle*, respectively.

**Fig. 6:**
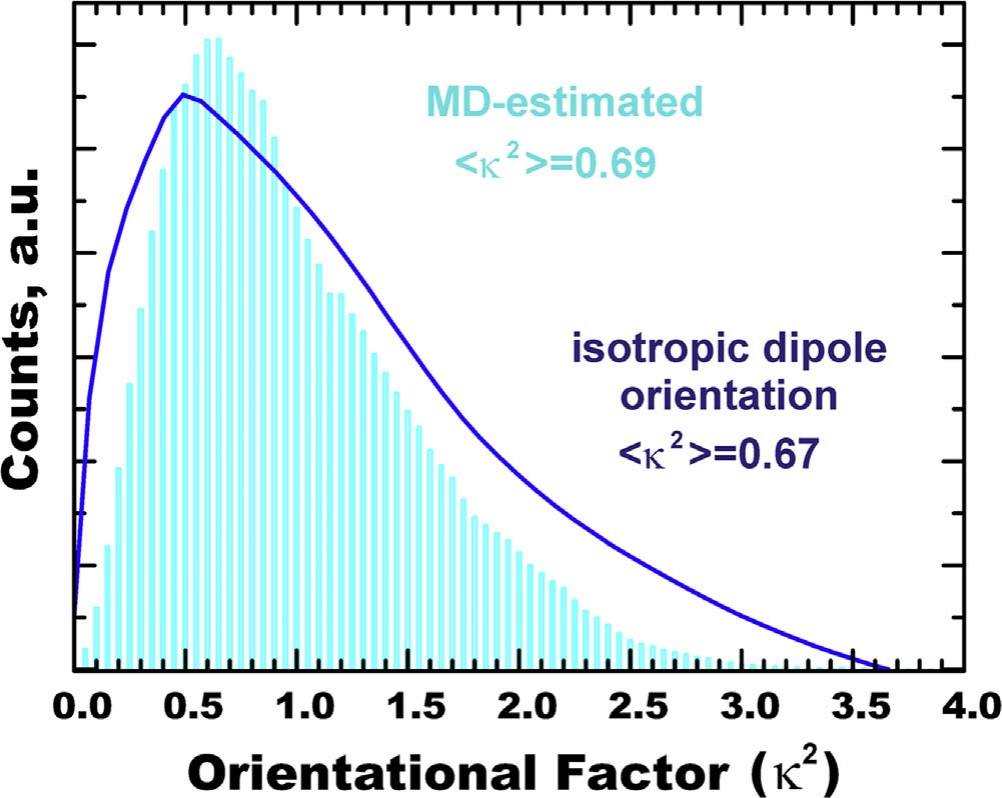
Comparison of the MD-simulated distribution of <*κ*^2^> (*cyan*) with the theoretical curve for an ideal unrestricted isotropic dynamic orientation of dipoles <*κ*^2^>=2/3 (*blue*).

The instantaneous orientation factor *κ*^2^(*t*) for the FRET ECFP-EYFP molecular pair was calculated from the instant position coordinates of the MD simulation trajectory, which allowed us to estimate the distribution of the donor and acceptor transition dipoles (Fig. 6). The MD-estimated average value of <*κ*^2^>=0.69 is in close agreement with the isotropic limit of 2/3, serving as an additional proof of sufficient conformational sampling. Comparison of the simulated distribution of *κ*^2^ with the theoretical angular distribution for isotropic dipoles in a three-dimensional system [6], [7] is shown in Fig. 6. While overall agreement was observed, it might be noted that the donor–acceptor pair linked by a series of chemical bonds still shows some minor deviations from ideal angular orientations of random non-interacting dipoles, also observed for other systems [8], [9], [10], [11].

### 1.4 MD-based calculation of FRET efficiency

The apparent FRET efficiency *E*_DA_ for the ECFP-l_3_-EYFP system was calculated from MD-generated time traces by using Eqs. (1), (2), the MD-estimated instantaneous value of the ECFP-to-EYFP distance *R*_DA,_ and the instantaneous orientation factor *κ*^2^(*t*), both evaluated at each MD trajectory step [1].

(1) $$E=\frac{1}{1+{\left(R_{DA}/R_{0}\right)}^{6}}$$

where *R*_0_ is the Förster radius characteristic for the specific dye pair involved in FRET. For ECFP/EYFP pair *R*_0_=48 Å [12], as calculated from the following

(2) $$R_{0}^{6}=\frac{9000\cdot (\ln\;10)\cdot \kappa^{2}Q_{D}}{128\cdot \pi^{5}Nn^{4}}J(\lambda )$$

where refractive index *n*=1.4 [12], [13], quantum yield of the donor *Q*_D^=^_0.4, the overlap integral *J(λ*) is calculated using the normalized ECFP emission spectrum and the EYFP excitation spectrum, normalized to 84,000 M^-1^ cm^-^^1^ at 514 nm. For *κ*^2^, a value of 2/3 was used, i.e., assuming dynamic averaging of the relative orientations of the ECFP emission and EYFP absorption dipole moments.

**Fig. 7:**
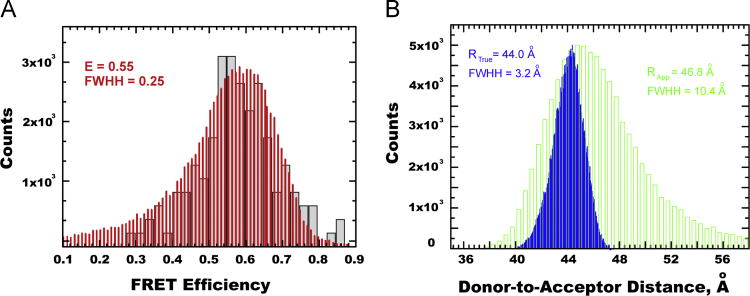
(**A**) Comparison of the experimentally observed *sm*-FRET efficiency (*grey bars*) and MD-reconstructed distribution of FRET probability. (**B**) Comparison of ECFP-to-EYFP distance distribution histograms calculated by the direct MD sampling (*blue*) and reconstructed from the MD-estimated *E*_DA_, under the standard simplifying assumption of unique *R*_0_=48 Å (*green*) (Raw data associated with this Figure are provided in Supplementary files).

The mean value of the MD-estimated apparent FRET efficiency was estimated to be 0.55 and the corresponding E_DA_ histogram reveals a broad distribution (FWHH=0.25), which can be compared to the distribution obtained from the *sm*-FRET measurements (Fig. 7A) [1].

### 1.5 Reconstructing *R*_DA_ distance distributions from FRET efficiencies

First, we used MD-generated FRET efficiency *E*_DA_ to reconstruct the “apparent” Donor–Acceptor distance distribution (*R*_App_), assuming a unique Förster distance of *R*_0_=48 Å that corresponds to the average orientational factor *κ*^2^=0.69 (Eq. 1). Then, we compared it to the “true” distance distribution (*R*_True_) directly estimated from the MD trajectory (Fig. 7B).

**Fig. 8:**
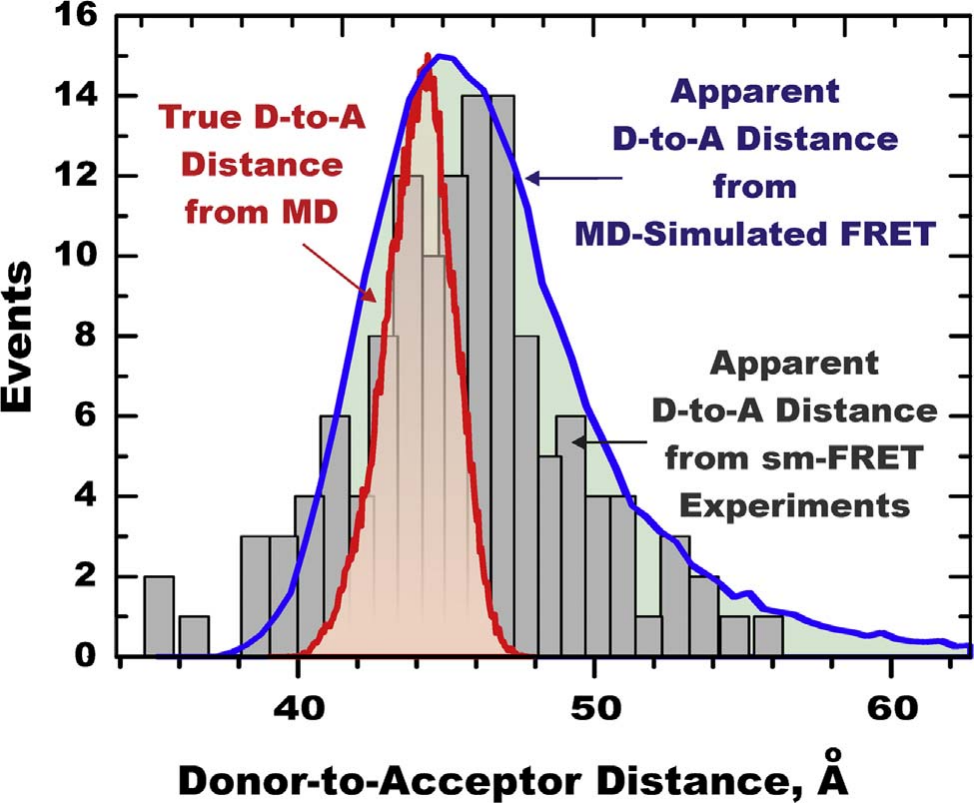
Comparison of True and Apparent distance distributions in ECFP-*l*_*3*_-EYFP model FRET system**.** True Donor-to-Acceptor distance distribution (*red*) is calculated directly from MD trace without any assumptions. Apparent Donor-to-Acceptor distance distributions are calculated from either experimental sm-FRET data (*black*) or MD-generated FRET data, assuming an average orientation factor <*κ*^2^>=0.69 (and subsequently unique value of *R*_0_=48 Å) for the entire population of conformations. (Raw data associated with this Figure are provided in Supplementary files).

The comparison of the effect of the standard assumption of the unique *R*_0_ value on the accuracy of determination of the Donor-to-Acceptor distance distributions is presented in Fig. 8. Both *apparent* distributions calculated from either MD-generated or experimentally measured FRET efficiencies, calculated under standard assumptions, systematically overestimate the width of the *true* distance distribution *R*_DA_. We conclude that a careful consideration of the orientational dynamics within a FRET pair is crucial for accurate measurements of distance distributions.

## 2 Experimental design, materials and methods

### 2.1 Materials

The materials and methods used to prepare the sample and collect the absorption, fluorescence intensity and fluorescence decay and sm-FRET data are given in [1], [14].

### 2.2 Molecular dynamics simulation setup

The structural model of peptide ECFP-l_3_-EYFP [12] was designed based on available X-ray structures of ECFP (PDB code: 1CV7) and EYFP (PDB code: 1YFP) [15], connected by flexible linker TLGMDELYKSGIR(GGSGGS)_3_-TMVS referred to as l_3_. The CHARMM27 force field for proteins, recently adopted for the GROMACS package, was used [16]. In this force field, the CHARMM27 CMAP correction term was implemented. The solvent water was modeled using the special CHARMM (TIP3P) model [16]. The bond length and angle parameters for a chromophore residue of ECFP and EYFP were optimized by density functional theory calculations at the B3LYP/cc-pVDZ level and adopted for the CHARMM27 force field. Partial charges needed for Coulomb interactions were derived from the B3LYP/ccpVDZ electron densities by fitting the electrostatic potential to point (ESP) charges [17]. The S_0_→S_1_ transition dipole moment needed for calculation of the orientation factor *κ*^2^ was derived from time-dependent density functional calculations TD-B3LYP/cc-pVDZ, and the corresponding dipole unit vectors are shown in Fig. 5. The estimated direction of the excited-state transition dipole moments of the ECFP and EYFP chromophores are found to be in agreement with those previously published for the chromopores of the green fluorescent protein family [18]. The MD topology file for polypeptide ECFP-l_3_-EYFP was built using GROMACS pdb2gmx utility. The CHARMM27 topology building blocks for non-native amino acid residues CRO and CRF were implemented using the corresponding GROMACS RTP library.

All MD simulations were carried out at a constant number of particles, constant pressure (*P*=1 atm), and constant temperature (*T*=298 K, NPT ensemble). Three-dimensional periodic boundary conditions were applied with the *z*-axis lying along a direction normal to the bilayer. The pressure was controlled isotropically, so that the *x, y* and *z* dimensions of the simulation box were allowed to fluctuate independently from each other, keeping the total pressure constant. The reference temperature and pressure were kept constant using the Berendsen weak coupling scheme with a coupling constant of *τ*_*T*_=0.1 ps for the temperature coupling and *τ*_*P*_=1.0 ps for the pressure coupling [19]. Electrostatic interactions were simulated with the particle mesh Ewald (PME) approach using the long-range cutoff of 0.8 nm [20]. The cutoff distance of Lennard-Jones interactions was also equal to 0.8 nm. All bond lengths in the protein were kept constant using the LINCS routine [21]. The MD integration time step was 2 fs. The MD simulations were carried out using the GROMACS set of programs, version 4.5.5 [22]. Molecular graphics and visualization were performed using VMD 1.8.6 software packages [23].

### 2.3 Molecular dynamics simulation flowchart

A series of MD simulation runs of ECFP-l_3_-EYFP, each starting with a different initial configuration, was performed according to the following flowchart: To initialize MD simulations, the FP protein was placed in a rectangular unit cell with a minimum distance of 2.0 nm from the box edge. The MD unit cell was filled with TIP3P water molecules by using GROMACS *genbox* utility. Next, steepest descent energy minimization was performed followed by an addition of ions in order to counterbalance the protein charge, yielding a final system of 11 Na^+^ ions. The control MD sampling was also performed in the presence of 0.1 M buffering ions Na^+^ and Cl^-^. Before accumulating MD conformational sampling, each starting system was taken through a 10 ns pre-equilibration run. To achieve better MD statistics, four initial configurations of ECFP-l_3_-EYFP with different distances and orientations between ECFP (donor) and EFYP (acceptor) were sampled. These four conformations of ECFP-l_3_-EYFP were modeled in MD unit cells of the following sizes: 149.5×149.5×149.5 Å (107902 waters), 202.4×102.1×102.1 Å (64973 waters), 116.2×116.2×116.2 Å (48461 waters) and 141.6×101.4×101.4 Å (45049 waters). The MD simulations ran for a total time of 1.5 μs, from which the last 500 ns were taken for the equilibrium FRET analysis.

## Supporting information

Raw simulation data of various conformations of protein ECFP-l3-EYFP are provided in PDB format. Supplementary raw data shown in Fig. 7, Fig. 8 can be found in the online version of the paper.

## Footnotes

Transparency document Transparency data associated with this article can be found in the online version at http://dx.doi.org/10.1016/j.dib.2017.03.041.

## References

[1] Kyrychenko A., Rodnin M.V., Ghatak C., Ladokhin A.S. (2017). Joint refinement of FRET measurements using spectroscopic and computational tools. Anal. Biochem..

[2] Caves L.S.D., Evanseck J.D., Karplus M. (1998). Locally accessible conformations of proteins: multiple molecular dynamics simulations of crambin. Protein Sci..

[3] Mirjalili V., Feig M. (2012). Protein structure refinement through structure selection and averaging from molecular dynamics ensembles. J. Chem. Theory Comput..

[4] Mirjalili V., Noyes K., Feig M. (2014). Physics-based protein structure refinement through multiple molecular dynamics trajectories and structure averaging, proteins: structure. Funct., Bioinforma..

[5] Kyrychenko A., Wu F., Thummel R.P., Waluk J., Ladokhin A.S. (2010). Partitioning and localization of environment-sensitive 2-(2′-pyridyl)- and 2-(2′-pyrimidyl)-indoles in lipid membranes: a joint refinement using fluorescence measurements and molecular dynamics simulations. J. Phys. Chem. B.

[6] Loura L.M.S. (2012). Simple estimation of Forster Resonance Energy Transfer (FRET) orientation factor distribution in membranes. Int. J. Mol. Sci..

[7] van der Meer B.W. (2002). Kappa-squared: from nuisance to new sense. Rev. Mol. Biotechnol..

[8] VanBeek D.B., Zwier M.C., Shorb J.M., Krueger B.P. (2007). Fretting about FRET: correlation between κ and R. Biophys. J..

[9] Deplazes E., Jayatilaka D., Corry B. (2011). Testing the use of molecular dynamics to simulate fluorophore motions and FRET. Phys. Chem. Chem. Phys..

[10] Hoefling M., Lima N., Haenni D., Seidel C.A.M., Schuler B., Grubmüller H. (2011). Structural heterogeneity and quantitative FRET efficiency distributions of polyprolines through a hybrid atomistic simulation and Monte Carlo approach. PLoS One.

[11] Walczewska-Szewc K., Corry B. (2014). Do bifunctional labels solve the problem of dye diffusion in FRET analysis?. Phys. Chem. Chem. Phys..

[12] Evers T.H., van Dongen E.M.W.M., Faesen A.C., Meijer E.W., Merkx M. (2006). Quantitative understanding of the energy transfer between fluorescent proteins connected via flexible peptide linkers. Biochemistry.

[13] Lakowicz J.R. (2006). Principles of Fluorescence Spectroscopy.

[14] Kyrychenko A., Posokhov Y.O., Rodnin M.V., Ladokhin A.S. (2009). Kinetic intermediate reveals staggered pH-dependent transitions along the membrane insertion pathway of the diphtheria toxin T-domain. Biochemistry.

[15] Wachter R.M., Elsliger M.-A., Kallio K., Hanson G.T., Remington S.J. (1998). Structural basis of spectral shifts in the yellow-emission variants of green fluorescent protein. Structure.

[16] Bjelkmar Pr, Larsson P., Cuendet M.A., Hess B., Lindahl E. (2010). Implementation of the CHARMM force field in GROMACS: analysis of protein stability effects from correction maps, virtual interaction sites, and water models. J. Chem. Theory Comput..

[17] Kyrychenko A., Ladokhin A.S. (2013). Molecular dynamics simulations of depth distribution of spin-labeled phospholipids within lipid bilayer. J. Phys. Chem. B.

[18] Ansbacher T., Srivastava H.K., Stein T., Baer R., Merkx M., Shurki A. (2012). Calculation of transition dipole moment in fluorescent proteins-towards efficient energy transfer. Phys. Chem. Chem. Phys..

[19] Berendsen H.J.C., Postma J.P.M., van Gunsteren W.F., DiNola A., Haak J.R. (1984). Molecular dynamics with coupling to an external bath. J. Chem. Phys..

[20] Darden T., York D., Pedersen L. (1993). Particle mesh Ewald: an Nx log(N) method for Ewald sums in large systems. J. Chem. Phys..

[21] Hess B., Bekker H., Berendsen H.J.C., Fraaije J.G.E.M. (1997). LINCS: a linear constraint solver for molecular simulations. J. Comput. Chem..

[22] Spoel D. Van Der, Lindahl E., Hess B., Groenhof G., Mark A.E., Berendsen H.J.C. (2005). GROMACS: fast, flexible, and free. J. Comput. Chem..

[23] Humphrey W., Dalke A., Schulten K. (1996). VMD: visual molecular dynamics. J. Mol. Graph..

